# Neuropsychophysiological Effects of a Single Functional Neurology Intervention on Semicircular Canals Stimuli Dysfunction

**DOI:** 10.3390/bs15030242

**Published:** 2025-02-20

**Authors:** Guillermo Escribano-Colmena, Jorge Rey-Mota, Vicente Javier Clemente-Suárez

**Affiliations:** 1 Independent Researcher, 28660 Boadilla del Monte, Spain; escribanocolmenaguillermo@gmail.com (G.E.-C.); jreymota@gmail.com (J.R.-M.); 2 Faculty of Sports Sciences, Universidad Europea de Madrid, Tajo Street, s/n, 28670 Madrid, Spain; 3 Grupo de Investigación en Cultura, Educación y Sociedad, Universidad de la Costa, Barranquilla 080002, Colombia

**Keywords:** functional neurology, semicircular canals, vestibular dysfunction, neuromuscular response, pain, cortical arousal

## Abstract

This exploratory observational study analyzed the neuropsychophysiological effects of Functional Neurology interventions on semicircular canal dysfunction, with a focus on enhancing neuromuscular responses and pain perception. A cohort of 45 healthy volunteers, comprising both males and females with an average age of 26.5 years, participated in this controlled, experimental study. The methodology involved baseline assessments of their psychophysiological state and physical abilities, followed by specific tests to analyze semicircular canal dysfunction. Participants then received customized Functional Neurology treatment aimed at correcting detected neuromuscular imbalances. The effects of Functional Neurology treatment were evaluated comparing variables such as hand strength, blood oxygen saturation, heart rate, and the Critical Flicker Fusion Threshold before and after the intervention. The study found a significant increase in the tolerance to semicircular canal stimuli, from an average of 1.0 ± 0.0 stimuli tolerated before intervention to 21.0 ± 14.0 post-treatment, suggesting that Functional Neurology can markedly improve neuromuscular responses in the context of vestibular dysfunction. However, no significant changes were observed in blood oxygen saturation or cortical arousal, indicating that these specific interventions may not affect all psychophysiological parameters. In conclusion, Functional Neurology interventions show promise in treating vestibular disorders by significantly enhancing neuromuscular response and pain tolerance, despite not impacting other psychophysiological measures. This research underscores the potential of Functional Neurology in improving the quality of life for individuals with vestibular dysfunctions and advocates for further exploration into its comprehensive neurophysiological effects.

## 1. Introduction

In the realm of neuroscience and vestibular rehabilitation, the semicircular canals are recognized for their pivotal role in maintaining balance and spatial orientation. These structures are integral to the vestibular system, enabling the detection of head movements and thus facilitating equilibrium and proprioception (Asadi et al., 2017). Research has advanced our understanding of the biomechanical responses of these canals, particularly in relation to vestibulo-ocular reflex (VOR) experiments, which illuminate how different head positions affect the semicircular canals’ response to angular movements (Fetsch et al., 2009). Dysfunctions within the semicircular canals can manifest in symptoms such as vertigo—specifically involving a false sense of rotational movement or spinning, often associated with inner ear disturbances—dizziness, which refers to a range of sensations such as feeling faint, unsteady, or weak without a false sense of motion, and imbalance, profoundly affecting an individual’s quality of life (Bloom, 2004). Vestibular disorders, including vestibular neuritis and benign paroxysmal positional vertigo (BPPV), often involve disruptions in the vestibular nerve’s transmission of signals from the semicircular canals to the brain, highlighting the complexity of the vestibular system and its critical role in sensory integration and balance maintenance (Goddard & Fayad, 2011).

Pathologies associated with dysfunctions in the semicircular canals are vast and varied, impacting balance, hearing, and overall quality of life. Conditions such as vestibular neuritis and labyrinthitis result from inflammation affecting the vestibular and possibly cochlear nerves, leading to symptoms like vertigo, hearing loss, and dizziness. These disorders underscore the complexity of the vestibular system and highlight the necessity for precise diagnosis and targeted treatment approaches. For instance, labyrinthitis may present with both vestibular and cochlear symptoms, necessitating treatments aimed at eradicating underlying infections and providing supportive care to alleviate symptoms (Thompson & Amedee, 2009). Moreover, vestibular disorders such as BPPV, Ménière’s disease, and vestibular migraine reveal varied patterns of semicircular canal and otolith involvement, suggesting distinct pathophysiological mechanisms. BPPV is characterized by brief episodes of vertigo triggered by head movements, and Ménière’s disease presents with episodic vertigo along with hearing loss and tinnitus, illustrating the diverse impact of vestibular disorders on the semicircular canals and otolith organs (Kim et al., 2022). BPPV is primarily an otolithic disorder, where dislodged otoliths (calcium carbonate crystals) from the utricle migrate into the semicircular canals. Although BPPV affects the semicircular canals by provoking abnormal fluid motion that leads to vertigo, it is fundamentally an otolithic issue rather than a direct disease of the semicircular canals themselves.

Functional Neurology emerges as a significant evolution in healthcare, particularly emphasizing the brain and nervous system’s adaptability—known as neuroplasticity—to enhance health outcomes. This field, rooted in chiropractic principles, positions nervous system functionality as pivotal to achieving and maintaining peak health and performance levels (Meyer & Leboeuf-Yde, 2018). Moving away from the conventional reliance on pharmaceuticals and surgeries, Functional Neurology instead opts for hands-on therapies and sensory-enriched interventions aimed at fostering neuroplastic changes (Møller, 2006). This shift acknowledges the nervous system’s lifelong capacity for adaptation and restructuring, challenging the earlier belief that such capabilities were confined to early development stages. At the heart of Functional Neurology lies the principle of neuroplasticity, allowing for the nervous system’s modification in response to tailored sensory, motor, or cognitive stimuli (Seidel-Marzi & Ragert, 2020). Practitioners leverage this dynamic capacity to manage and rectify neurological impairments through precise, targeted treatments, eliminating the need for medicinal or surgical interventions (Meyer et al., 2017). This approach is particularly relevant in environments demanding high performance, where maintaining optimal neurological function is essential for success. Functional Neurology underscores the critical role of balance, vestibular functions, and proprioception, which are essential for executing movements with precision and efficiency (Meyer & Leboeuf-Yde, 2018).

Functional Neurology, rooted in the principles of neuroplasticity, has shown promise in enhancing neuromuscular and proprioceptive functions. Studies have demonstrated its application in various contexts, including sensorimotor rehabilitation (Seidel-Marzi & Ragert, 2020) and postural control (Meyer et al., 2017). However, it has been highlighted that the evidence base for Functional Neurology remains limited, necessitating further research to validate its efficacy as an evidence-based treatment modality (Meyer & Leboeuf-Yde, 2018). This study seeks to contribute to this growing body of knowledge by exploring its effects on vestibular-related neuromuscular responses. While previous reviews, such as those by Meyer and Leboeuf-Yde (2018), have raised valid concerns about the lack of robust evidence supporting the benefits of Functional Neurology, this study aims to address these gaps by providing preliminary data on its neurophysiological effects. By leveraging controlled conditions and detailed neuromuscular assessments, this research contributes to establishing a more comprehensive understanding of the potential applications of Functional Neurology.

The main aim of our study was to analyze the psychophysiological changes following Functional Neurology interventions in cases of semicircular canal dysfunction. Central to our vestibular system, the semicircular canals are integral in maintaining balance and spatial awareness. Dysfunction within these canals can manifest as significant disruptions in neuromuscular coordination, balance, and pain perception. This study employed an exploratory observational design to analyze the effects of Functional Neurology interventions, acknowledging the absence of a control group and uniform treatments across participants to assess their capacity to effectuate alterations in physiological and sensory responses. Specifically, we concentrate on the modulation of pain perception, neuromuscular performance, cortical arousal, blood oxygen saturation, and heart rate as a marker of treatment efficacy. Our hypothesis posits that by addressing the aberrations in the semicircular canals, such interventions can substantially modulate an individual’s psychophysiological state, potentially enhancing neuromuscular coordination and mitigating pain, thereby contributing to an improved quality of life and functional performance. Although the participants had no history of vestibular disorders, the study aimed to explore the neurophysiological responses to semicircular canal stimuli in healthy individuals, as such responses may reveal subclinical neuromuscular imbalances. These imbalances, while not overtly symptomatic, could provide insights into the initial stages of vestibular dysfunction, supporting the hypothesis that Functional Neurology interventions could mitigate latent sensitivities and enhance neuromuscular coordination. Testing the vestibular function in healthy participants allows for the identification of adaptive limits in neuromuscular control and proprioceptive reflexes under semicircular canal stimulation. While such tests do not equate to diagnosing vestibular disorders, they provide a controlled framework for assessing the efficacy of Functional Neurology interventions in addressing potential neuromuscular imbalances associated with subclinical vestibular dysfunctions.

## 2. Materials and Methods

### 2.1. Participant

In this study, we analyzed 42 volunteer participants, of whom 9 were females and the remaining 33 were males. The cohort had an average age of 26.5 ± 5.0 years, a mean height of 177.0 ± 7.5 cm, a mean weight of 74.9 ± 12.4 kg, and an average body mass index (BMI) of 23.8 ± 2.9. To participate in this study, individuals had to meet specific inclusion and exclusion criteria designed to control for variables that might influence the outcomes. The inclusion criteria required participants to be healthy adults aged more than 18 years, with no history of vestibular disorders, neurological conditions, or psychiatric illnesses. Participants were screened to ensure they were not taking any medications that could affect the central nervous system. They were required to abstain from alcohol consumption and refrain from engaging in vigorous physical activity for at least 48 h before the study. Furthermore, participants were asked to fast for at least two hours before each testing session to standardize metabolic states. The exclusion criteria ruled out any individuals with a history of otological surgeries, such as cochlear implants or other invasive inner ear procedures, as these could directly affect the function of the semicircular canals. Participants with diagnosed vestibular disorders (e.g., benign paroxysmal positional vertigo, Ménière’s disease), chronic migraines, psychological disorders (e.g., clinical anxiety, depression), or any other chronic illnesses that might affect neuromuscular function were excluded. Additionally, recent head trauma or concussion history was a disqualifying factor. These exclusion criteria ensured that the study sample was homogenous and that any observed effects could be directly linked to the Functional Neurology intervention without the confounding influence of pre-existing health conditions. The study adhered to the ethical standards laid out in the Helsinki Declaration and received ethical approval from the University’s Bioethics Committee under code 2024-736.

### 2.2. Procedure

To fulfill the objectives of our study, we assessed the responses of each participant at four distinct time points to gain a detailed understanding of how the Functional Neurology intervention influenced their psychophysiological and muscular performance. The assessments were scheduled as follows:

1. Baseline Measurement: Initially, we conducted comprehensive evaluations to establish a baseline for each participant’s psychophysiological state and physical abilities. This initial phase acted as the control setting, providing essential baseline data for comparison with future assessments.
2. Pre-Intervention Indicator Muscle Failure: After the initial baseline measurements, a specific test was administered to trigger failure in a targeted muscle, namely the anterior deltoid, to observe its natural neurological reaction to semicircular canal stimuli. The assessments taken immediately following the muscle’s failure to sustain performance provided insights into the neurophysiological and muscular reactions to immediate stress prior to any treatment (less than 30 s). Muscle failure in the anterior deltoid was induced through a standardized isometric resistance test. Participants were instructed to hold an isometric contraction against gradually increasing resistance applied manually by a certified practitioner. The resistance was incrementally increased until the participant was unable to sustain the contraction for at least 3 s or exhibited compensatory activation of adjacent muscle groups, such as the pectoralis major or trapezius. Muscle failure was recorded when the participant’s effort failed to maintain proper alignment or contraction stability despite maximal effort. This method ensures consistent application and replicability of the protocol across participants (Rey-Mota et al., 2024a).
3. Functional Neurology Treatment: Participants received a custom Functional Neurology treatment following the induced muscle failure, aimed at correcting detected neuromuscular imbalances. This treatment was intended to optimize the body’s neuroplasticity for improved neural and muscular functioning. Measurements taken after this intervention allowed for the evaluation of immediate changes resulting from the Functional Neurology techniques (Rey-Mota et al., 2024b). The full treatment protocol is explained in Section 2.4.
4. Post-Intervention Indicator Muscle Failure: The concluding evaluations were performed after repeating the semicircular canal stimuli test to induce muscle failure again, this time to observe the neurological response of the muscle post-treatment. These final assessments were crucial for assessing the treatment’s impact on enhancing the muscle’s resilience and the overall neuromuscular health of the participants. During the second measurement period as well as in the first one, muscle failure in the anterior deltoid was induced using a targeted resistance test. Participants were instructed to maintain an isometric contraction of the anterior deltoid against gradually increasing resistance applied manually by a certified practitioner. The test continued until the participant was unable to sustain the contraction for at least 3 s or exhibited visible compensation from other muscle groups, such as the trapezius or pectoralis major. To ensure consistency and minimize subjectivity, all tests were conducted by the same practitioner with over five years of experience in manual muscle testing. Additionally, a standardized protocol was followed, including verbal encouragement and precise hand positioning to ensure consistent resistance application. Muscle failure was defined as the point at which the anterior deltoid could no longer sustain contraction despite maximal effort, as confirmed by the practitioner and recorded in the participant’s assessment log (Rey-Mota et al., 2024a, 2024b).

All evaluations were carried out in a controlled environment, with a consistent temperature of 23.1 ± 0.5 °C and humidity of 40.7 ± 3.0%. All assessments were conducted between 8:00 AM and 11:00 AM to reduce the impact of diurnal variations on the measured physiological parameters. This scheduling aimed to minimize the influence of circadian rhythms on outcomes such as hand strength, heart rate, blood oxygen saturation, and the critical flicker fusion threshold. While this approach helped to standardize testing conditions, we acknowledge that some degree of diurnal variability may still have affected the results. Future studies could consider using repeated measures at different times of the day to better understand the impact of diurnal fluctuations on the efficacy of Functional Neurology interventions.

### 2.3. Instruments and Study Variables

In our investigation, we adhered to established tools and methodologies for collecting the relevant data as detailed below:

- Body weight was measured using a SECA scale (model 714, SECA GmbH & Co. KG, Hamburg, Germany), which offers a precision of up to 100 g and is capable of measurements ranging from 0.1 to 130 kg. Prior to use, the scale was calibrated to zero and positioned on a hard, level surface. Participants were asked to disrobe any heavy clothing and footwear, standing centrally on the scale’s platform, ensuring they were upright, not touching any nearby objects, and distributing their weight equally across both feet (Tornero-Aguilera et al., 2022).
- The Pressure Pain Threshold (PPT) was determined using a manual pressure algometer, the FPK 60 model from Wagner Instruments Inc., Greenwich, CT, USA. The device was applied vertically to the muscle area, increasing pressure steadily at a rate of 1 kg/s until the participant indicated the onset of pain. This measure was recorded at designated points on the upper body, specifically the midpoint of the upper trapezius muscle’s anterior edge, focusing on the vertical fibers near the clavicle (Zamani et al., 2017).
- Cortical arousal was assessed by the Critical Flicker Fusion Threshold (CFFT) using a device contained within a viewing chamber, the Lafayette Instrument Flicker Fusion Control Unit (Model 12021, Lafayette Instrument Company, Lafayette, IN, USA). An elevated CFFT indicates an increase in cortical arousal and information processing efficiency, whereas a decline suggests a decrease in these capacities, potentially indicating central nervous system fatigue (Belinchon-deMiguel et al., 2023).
- Isometric hand-grip strength was measured with a TKK 5402 dynamometer (Takei Scientific Instruments Co. Ltd., Niigata, Japan). For this assessment, participants were seated with their arm positioned at a 90-degree angle and their forearm in a neutral stance. The best score from two attempts was recorded for the dominant hand (Hormeño-Holgado & Clemente-Suárez, 2019).
- Blood oxygen saturation and the heart rate were monitored using a Beurer PO 30 pulse oximeter (Beurer GmbH, Ulm, Germany). These parameters were tracked to evaluate the physiological impact of the intervention and to gather insights into the cardiovascular system’s functionality during the tests (Hormeño-Holgado et al., 2019; Sánchez-Molina et al., 2019).
- For the semicircular canal stimuli test utilized in our study, participants were instructed to keep their heads stationary while following a finger with their eyes. We analyzed the number of stimuli tolerated until indicator muscle failure. The number of semicircular canal stimuli tolerated until dysfunction in an indicator muscle (anterior deltoid) was measured during the semicircular canal stimuli test. Each stimulus was defined as a complete sequence of eye-tracking movements guided by the practitioner, as described earlier. The test continued until the participant exhibited muscle dysfunction, defined as an inability to sustain contraction against resistance, visible compensation from adjacent muscle groups, or failure to complete subsequent stimuli. The total number of completed stimuli prior to dysfunction was recorded as the metric for tolerance. If there was any dysfunction in the vestibular system, an inhibitory response of the muscle reflexes was triggered. This response was evaluated through the indicator muscle test, allowing us to identify vestibular system dysfunction using this muscular assessment (Rey-Mota et al., 2024b).
- Head rotation speed and the measurement of eye movement direction and speed were standardized to ensure consistency across participants. Participants were instructed to perform smooth-pursuit eye tracking while maintaining their heads stationary at a neutral position. The practitioner moved a target (finger) at a fixed speed of approximately 20 cm per second along pre-defined trajectories: horizontal, vertical, and diagonal directions. This speed was chosen based on prior research indicating optimal activation of semicircular canal reflex pathways under these conditions. Eye movements were visually monitored by the practitioner, who ensured adherence to the fixed target speed and direction. While we did not employ high-precision tracking devices to measure eye movement speed quantitatively, the consistency of manual guidance and practitioner experience minimized variability in the test execution. Future studies may benefit from incorporating video-based eye-tracking systems or vestibular function testing tools to enhance measurement precision and allow for a more detailed analysis of eye movement dynamics (Mori & Katayama, 2005; Rey-Mota et al., 2025).
- Diagonal eye movements are complex actions that require the coordinated activation of multiple sensory systems, including the vestibular system. In particular, the semicircular canals of the vestibular system play a crucial role in gaze stabilization during these movements. The semicircular canals are sensory structures in the inner ear that detect angular acceleration of the head in three perpendicular planes, corresponding to rotational movements along the horizontal, vertical, and diagonal axes. When the head moves in a diagonal direction, these canals send signals to the brain to activate the vestibulo-ocular reflex (VOR), which generates compensatory eye movements in the opposite direction to maintain visual stability. During diagonal tracking, the activation of the semicircular canals is uniquely combined, as these movements involve both horizontal and vertical components. This means that more than one semicircular canal is activated simultaneously, requiring more complex integration between vestibular and visual stimuli.

This integration is essential to keep the image stable on the retina during diagonal movements, which is crucial for tasks requiring visual precision, such as reading a screen or following a moving object. Studies have shown that diagonal tracking movements can also be influenced by the visual environment and the speed of head rotation. For example, in situations where the head performs rapid diagonal movements, the activation of the semicircular canals may lead to quick adjustments in the direction and speed of eye movements, ensuring that visual perception remains stable.

In summary, diagonal tracking and the activation of the semicircular canals are closely related through the VOR, allowing the vestibular and visual systems to work together to stabilize the gaze and ensure clear visual perception during complex head movements (Lopez, 2015).

### 2.4. Functional Neurology Intervention

In our research, participants underwent a customized Functional Neurology treatment, incorporating blink reflex techniques, drawing from the exclusive methodologies of NeuroReEvolution^®^ (http://nre-therapy.com/, accessed on 15 January 2025), a practice characterized by its unique specialization. The initial step of this individualized therapy involved a comprehensive clinical assessment, encompassing both verbal and visual evaluations, as well as specific Functional Neurology examinations of joints, to pinpoint the precise neurological issues of each participant. A certified professional, equipped with a Level III certification in the Functional Neurology Manual Muscle Test from NeuroReEvolution^®^, leveraged this deep understanding to tackle the distinct neurological imbalances found in each participant, ensuring a highly personalized and effective therapeutic strategy (Rey-Mota et al., 2024b, 2025).

The essence of Functional Neurology treatment revolves around its emphasis on enhancing proprioceptive reflexes, alleviating trigger point sensitivity, and adopting a comprehensive, systemic perspective. Proprioceptive reflexes, the nervous system’s inherent reactions to physical changes like stretching muscles or compressing tendons, are vital for the regulation of posture, equilibrium, and locomotion. The therapeutic intervention targeted anomalies in these reflexes that could lead to discomfort or limit movement.

The therapy precisely located any dysfunctional mechanoreceptor and/or nociceptor. Through manual intervention and specific stimuli of the mechanoreceptors and nociceptors, the treatment aimed to reduce the sensitivity of these points, thus adjusting the nervous system’s response to diminish discomfort and support functional rehabilitation.

Adopting a holistic perspective, this method considered the body as a fully integrated entity, recognizing that issues in one region could affect remote areas via the nervous system’s reflex network. This approach sought to remedy not just the localized dysfunction but also any associated conditions that might affect the individual’s comprehensive neuromuscular well-being (Rey-Mota et al., 2024a).

Neuromuscular imbalances were identified through manual muscle testing, reflex evaluations, and proprioceptive assessments conducted by certified Functional Neurology practitioners. These imbalances often manifested as altered reflex thresholds or asymmetries in proprioceptive responses. Customized treatments included targeted proprioceptive recalibration, manual interventions to reduce trigger point sensitivity, and eye-tracking exercises designed to optimize neuroplasticity. While these treatments were tailored to individual participant profiles, the overarching approach sought to standardize the correction of detected anomalies in vestibular and proprioceptive systems.

### 2.5. Statistical Analysis

In our research, the data analysis was conducted using version 20.1 of the IBM SPSS Statistics software. To summarize the data, means and standard deviations (SDs) were calculated for each variable. We checked the distribution of the data for normality with the Kolmogorov–Smirnov test. Once the data were confirmed to follow a normal distribution, we proceeded with parametric statistical tests. A one-way repeated-measures ANOVA was applied to analyze changes in outcome variables across the four time points, followed by Bonferroni-adjusted post hoc tests to account for multiple comparisons. This approach ensured the robust identification of statistically significant differences while minimizing Type I errors. We considered findings with a *p*-value of 0.05 or lower as statistically significant, indicating a meaningful difference or effect.

## 3. Results

The Functional Neurology intervention led to significant improvements in several measured variables. Participants demonstrated an increase in the number of semicircular canal stimuli tolerated, from an average of 1.0 (SD = 0) before treatment to 21.0 (SD = 14.0) post-treatment (F = 91.603, *p* < 0.001, ηp^2^ = 0.676). Hand grip strength increased significantly between Times 1 and 3, as well as Times 2 and 3, with the largest difference being 5.4 units (F = 9.150, *p* < 0.001, ηp^2^ = 0.413). Significant reductions in the heart rate were observed post-treatment compared to the baseline (*p* = 0.005) and post-muscle failure (*p* = 0.039). Detailed statistical data for all variables are summarized in Table 1.

## 4. Discussion

The findings of this exploratory observational study provide preliminary evidence of the effects of Functional Neurology interventions on neuromuscular performance and pain perception. However, the absence of a control group, the lack of follow-up assessments, and the individualized nature of treatments highlight the need for further research with more rigorous experimental designs. The objective of this research was to analyze the psychophysiological changes elicited by Functional Neurology interventions in individuals with dysfunctions of the semicircular canals. In addressing our hypothesis, the data indicate that such interventions have a measurable impact, showing that restoration of semicircular canal function can lead to enhanced neuromuscular responses and a reduction in pain perception, thereby underlining the potential of Functional Neurology in the treatment of vestibular dysfunctions. The psychophysiological improvements noted in our study after Functional Neurology (FN) interventions are reflected in the broader context of the vestibular rehabilitation literature. These improvements are consistent with the proposition that active behavioral engagement is necessary to start the vestibular compensation process. Such an approach can enhance the central nervous system’s sensorimotor cues and regulate vestibular functions, including posture and gaze stabilization (Lacour & Bernard-Demanze, 2015). Moreover, vestibular disorders have been linked to cognitive challenges, underlining the potential cognitive benefits of FN interventions in our participants (Smith et al., 2024).

The Functional Neurology intervention’s substantial effect on enhancing tolerance to semicircular canals stimuli is indicated by the increased number of stimuli tolerated by the participants. This marked improvement not only underscores the intervention’s effectiveness in addressing semicircular canal dysfunction but also highlights the potential for Functional Neurology to foster notable neuroplastic changes, enhancing the neuromuscular coordination and function (Meyer et al., 2017). This aligns with the concept that targeted sensory and motor stimuli can induce neuroplasticity, facilitating the restoration of vestibular functions and improving overall neuromuscular health. The magnitude of change observed, coupled with the high effect size, suggests that the interventions employed could be particularly beneficial for individuals experiencing vestibular dysfunctions, potentially leading to significant improvements in quality of life and functional performance.

Our findings suggest that Functional Neurology interventions have the potential to provide cognitive and neuromuscular benefits even in asymptomatic individuals. Despite our participants being healthy and without documented vestibular dysfunction, the total inhibition of body musculature observed when exposed to a single semicircular canal stimulus indicates a latent sensitivity that may not yet manifest as overt vestibular symptoms. This latent sensitivity could represent an early, subclinical phase that predisposes individuals to more pronounced vestibular-related pathologies and symptoms in the future. By improving neuromuscular response and enhancing tolerance to vestibular stimuli, FN interventions may play a preventative role, stabilizing vestibular function and reducing the likelihood of progression to more severe vestibular dysfunctions. This stabilization not only supports neuromuscular coordination but may also mitigate cognitive challenges often associated with vestibular disorders, such as difficulties with spatial orientation, memory, and attention. The potential for FN interventions to preemptively address these issues underscores their value in a broader context of health and wellness, beyond treating individuals with overt vestibular pathologies. Future research should focus on the long-term cognitive and neuromuscular benefits of FN interventions in asymptomatic populations, examining their role in preventing the onset of vestibular dysfunctions and associated cognitive decline. This approach could provide insights into optimizing preventive healthcare strategies and improving the quality of life for individuals at risk of developing vestibular disorders (Rey-Mota et al., 2024a, 2024b).

Nevertheless, we found that the interventions applied did not yield significant changes in Blood Oxygen Saturation or cortical arousal as determined by the Critical Flicker Fusion Threshold. This outcome suggests a nuanced understanding of how Functional Neurology interventions influence the physiological aspects underpinning vestibular disorders. The stability of BOS throughout the intervention might indicate that the short-term impact of these treatments does not extend to systemic oxygenation levels, which could be attributed to the nature and immediacy of the interventions used. Similarly, the unchanged CFFT scores post-intervention may reflect a more complex relationship between vestibular functions and cortical arousal processes than previously understood. This contrasts with other studies in which acute high-intensity exercise interventions did lead to a decrease in cortical arousal, such as tennis high-intensity interval training (Clemente-Suárez et al., 2022), and notably in cardiac patients undergoing high-intensity interval training (Gonçalves et al., 2022) or military personnel facing simulated combat situations (Tornero-Aguilera et al., 2021).

The improvements in hand strength and heart rate modulation in our subject’s post-treatment can be associated with the evidence suggesting that vestibular signals contribute to proprioception and the regulation of motor functions (Lopez, 2015). These findings are consistent with recent literature demonstrating that interventions targeting vestibular structures have implications for both motor and cognitive domains, underlying the interconnectedness of vestibular signals with widespread brain functions (St George & Fitzpatrick, 2011). The enhanced hand strength and heart rate stabilization noted in our study likely reflect the central role of the vestibular system in motor function regulation, as supported by the literature. Vestibular inputs are crucial not only for maintaining balance but also for coordinating movements and orienting oneself in space (Lacour, 2006). This multisensory integration is essential for activities ranging from simple tasks to complex athletic performances, and dysfunctions can significantly impact motor control and cognitive functions like spatial orientation (Møller, 2006). Moreover, recent findings suggest that vestibular rehabilitation can lead to neuroplastic changes in the brain, contributing to improved motor and cognitive outcomes (Rogge et al., 2018). This is supported by previous research (Lee et al., 2022), in which it was found that certain vestibular rehabilitation maneuvers can influence recovery rates in horizontal semicircular canal cupulolithiasis.

The study observed statistically significant increases in hand strength between Times 1 and 3 and Times 2 and 3, with the largest difference being 5.4 units. While these differences were statistically significant, their practical or clinical relevance remains uncertain. A change of 5.4 units in hand strength may not represent a meaningful improvement in functional capacity for most individuals. Future studies should aim to establish thresholds for minimal clinically important differences in hand strength, particularly in populations with specific functional impairments, to better contextualize the observed changes. Additionally, incorporating functional assessments or task-specific performance measures could provide further insight into the real-world implications of these findings. While several statistically significant effects were observed in this study, it is important to recognize that statistical significance does not inherently imply a meaningful or clinically relevant difference. For instance, the observed increase in hand grip strength, though statistically significant, involved a change of only 5.4 units at its largest. This magnitude of change may not translate to substantial functional or practical improvements for individuals in real-world settings. Future studies should consider incorporating measures of effect size and minimal clinically important differences to contextualize the relevance of observed changes and provide a more comprehensive assessment of the intervention’s practical impact.

Our results, demonstrating a significant improvement in pain perception and neuromuscular response, complement recent advances in the therapeutic approaches for vestibular disorders, which include not only physical maneuvers but also consideration of the cognitive and emotional status of patients (Oka et al., 2023). This aligns with Pérez-Fernández and Ramos-Macías (2023), who emphasize the expansive scope of vestibular medicine, acknowledging the complexity of vestibular symptoms that span from the inner ear to cerebral networks. The efficacy of such interventions is further supported by evidence that indicates compensatory processes play a role in the resolution of vestibular symptoms and the restoration of function, as observed in longitudinal studies of patients with vestibular neuritis (Esteban-Sánchez & Martín-Sanz, 2022). The notable improvement in pain tolerance observed in our study is consistent with the advancements in the field of vestibular disorder treatments, which emphasize a holistic approach that incorporates physical, cognitive, and emotional rehabilitation strategies (Pérez-Fernández & Ramos-Macías, 2023). Innovations such as vestibular implants for bilateral vestibulopathy (Merchant et al., 2005) and new therapeutic maneuvers for semicircular canal cupulolithiasis (Lee et al., 2022) reflect the evolving landscape of less invasive yet effective treatments. Additionally, longitudinal analyses highlight the dynamic nature of vestibular compensation and its association with improved postural control and reduced pain perception (Esteban-Sánchez & Martín-Sanz, 2022), while research into the psychophysics of vestibular disorders sheds light on the complex relationship between vestibular function and pain tolerance (Kayan & Hood, 1984; Lempert & Neuhauser, 2009). These findings underscore the importance of integrated treatment modalities that not only aim to restore physical balance but also enhance pain tolerance and overall quality of life. Finally, these changes indicate variability in pain perception that cannot be definitively attributed to the Functional Neurology intervention. Without a control group, the reduction in pain thresholds may result from repeated testing, participant habituation, or other confounding factors. Future studies should include randomized controlled designs to isolate the specific effects of the intervention on pain perception.

We found how the interventions applied did not yield significant changes in blood oxygen saturation or cortical arousal as determined by the Critical Flicker Fusion Threshold. This outcome suggests a nuanced understanding of how Functional Neurology interventions influence the physiological aspects underpinning vestibular disorders. The stability of BOS throughout the intervention might indicate that the short-term impact of these treatments does not extend to systemic oxygenation levels, which could be attributed to the nature and immediacy of the interventions used. Similarly, the unchanged CFFT scores post-intervention may reflect a more complex relationship between vestibular functions and cortical arousal processes than previously understood. These outcomes may reflect the specificity of the intervention, which primarily targeted neuromuscular and proprioceptive systems rather than broader physiological or cognitive processes. Alternatively, the measures used may not have been sensitive enough to detect subtle changes in these parameters. Further research with more sensitive or alternative assessment tools is necessary to explore these aspects in greater detail. This contrasts with other studies in which acute high-intensity exercise interventions did lead to a decrease in cortical arousal, such as tennis high-intensity interval training (Clemente-Suárez et al., 2022), and notably in cardiac patients undergoing high-intensity interval training (Gonçalves et al., 2022) or military personnel facing simulated combat situations (Tornero-Aguilera et al., 2021).

The study population consisted of healthy young adults with a mean age of 26.5 years, which limits the generalizability of the findings to clinical populations with vestibular dysfunction. While the use of healthy participants allowed for controlled exploration of neuromuscular responses and subclinical vestibular sensitivities, the results may not reflect the effects of Functional Neurology interventions in individuals with diagnosed vestibular disorders or in older populations with age-related vestibular impairments. Future studies should include diverse populations with varying degrees of vestibular dysfunction to better assess the clinical relevance of these interventions.

### 4.1. Practical Applications

Our study provides valuable insights into the potential benefits of Functional Neurology interventions for vestibular dysfunction, particularly in enhancing pain perception and neuromuscular response, suggesting its viability as part of a comprehensive treatment approach (Pérez-Fernández & Ramos-Macías, 2023). However, the absence of significant changes in cortical arousal and blood oxygen saturation calls for further investigation into the interventions’ broader physiological impacts and the potential need for longer-term or varied approaches. This underscores the importance of a multidisciplinary and nuanced treatment strategy for vestibular disorders, taking into account the physical, cognitive, and emotional aspects of patient care, and highlights the need for continued research into optimizing treatment modalities for these conditions (Rey-Mota et al., 2024b).

While Functional Neurology interventions have demonstrated potential benefits in neuromuscular response and pain perception, their clinical application requires careful consideration. Individualized assessments are necessary to determine the suitability of the intervention for each patient, particularly in cases of severe vestibular dysfunction. Clinicians should be aware that responses to treatment may vary, and further studies are needed to establish standardized protocols. Additionally, caution should be exercised when applying these interventions to individuals with underlying neurological or cardiovascular conditions, as the impact on systemic physiological functions remains underexplored.

### 4.2. Study Limitations and Future Research Lines

This study reveals promising potential for Functional Neurology interventions in treating semicircular canal dysfunction but is subject to several limitations. The most critical limitation is the absence of a control group, which restricts the ability to isolate the true effects of the intervention. Additionally, the short-term nature of the study prevents capturing long-term neuroplastic adaptations. Another key limitation is the small and homogeneous participant cohort, which limits the generalizability of the findings to broader clinical populations. Furthermore, the lack of standardized vestibular assessment tools (such as caloric tests, vHIT, or VEMPs) reduces the clinical applicability of the results. A further limitation is that, while significant changes were observed in neuromuscular response and pain perception, no notable effects were found in blood oxygen saturation or cortical arousal, suggesting that the intervention may have selective physiological effects. Future research should address these limitations through larger sample sizes, the inclusion of diverse populations, and integration of advanced vestibular diagnostics. While this study provides preliminary evidence of Functional Neurology interventions’ effects, its findings are based on a sample of healthy young adults, which limits generalizability to older individuals or those with diagnosed vestibular disorders. Future research should include clinical populations with varying degrees of vestibular dysfunction to assess the intervention’s applicability in real-world therapeutic settings.

A key limitation of this study is the absence of follow-up assessments to determine the long-term effects of the intervention. Neuroplastic changes often develop progressively, and evaluating outcomes at multiple time points post-intervention would provide a clearer understanding of the intervention’s sustainability. Future research should incorporate longitudinal designs with repeated assessments to track the persistence of neuromuscular adaptations and pain tolerance over time. Some assessments, such as manual muscle testing and pain threshold reporting, rely on subjective measures, which may introduce variability and potential bias. Although standardization of procedures minimized this effect, future studies should incorporate objective neuromuscular and vestibular assessments, such as electromyography, force plate analysis, and quantitative sensory testing, to enhance measurement accuracy and reproducibility.

Future research should address these limitations by employing larger, more diverse participant samples, integrating standard neuro-otology assessment protocols, and using crossover or randomized controlled designs. Long-term follow-up studies are necessary to assess the sustainability of observed effects and their clinical relevance, particularly in populations with documented vestibular dysfunctions. Combining Functional Neurology with other rehabilitation modalities and incorporating more sensitive evaluation tools could further enhance understanding and optimization of treatment strategies.

Future research should focus on investigating the long-term effects of Functional Neurology interventions on neuroplasticity. Longitudinal studies with extended follow-up periods will be essential to assess whether the observed neuromuscular improvements are sustained over time. Additionally, randomized controlled trials in clinical populations with documented vestibular dysfunction should be conducted to determine the efficacy of these interventions beyond healthy individuals. It would also be beneficial to explore how Functional Neurology interacts with other vestibular rehabilitation techniques, such as balance training and cognitive therapy, to develop a more comprehensive therapeutic approach.

## 5. Conclusions

The results of this study suggest that Functional Neurology interventions may enhance tolerance to stimuli in the semicircular canals, indicating a potential for improving certain aspects of vestibular dysfunction. Specifically, the intervention demonstrated significant improvements in neuromuscular response and pain tolerance, which are critical factors in the management of vestibular disorders. However, no significant changes were observed in blood oxygen saturation or cortical arousal, indicating that these interventions may not influence all psychophysiological parameters related to vestibular function. While the study demonstrates promising neuromuscular and pain perception improvements following Functional Neurology interventions, these results should be interpreted cautiously. The findings highlight the potential for addressing subclinical vestibular sensitivities, but further research with clinical populations and appropriate control groups is necessary to substantiate the broader applicability of these treatments.

## Figures and Tables

**Table 1:** Impact of Functional Neurology intervention on psychophysiological and physical performance measures.

Variable	1	2	3	4	F Lambda de Wilks	Sig	ηp^2^	Post Hoc Comparison
PPT (kgf)	11.2 ± 10.0	8.8 ± 5.9	7.3 ± 3.0	11.1 ± 11.3	3.194	0.034	0.197	
Hand Strength (N)	38.7 ± 14.1	40.5 ± 12.4	44.1 ± 7.0	39.5 ± 12.9	9.150	<0.001	0.413	1 < 3 (0.017) 3 > 4 (0.029) 2 < 3 (0.012)
CFFT (Hz)	35.8 ± 3.3	35.5 ± 2.6	36.0 ± 2.9	35.8 ± 3.0	1.970	0.134	0.131	
Blood Oxygen Saturation (%)	96.5 ± 1.1	96.5 ± 1.3	96.4 ± 0.7	96.6 ± 1.5	0.496	0.687	0.037	
Heart Rate (bpm)	82.6 ± 16.2	80.6 ± 12.6	77.7 ± 15.4	78.3 ± 14.3	4.361	0.010	0.251	1 > 3 (0.005) 1 > 4 (0.039) 2 > 3 (0.021)

Baseline Measurement (1); Post-Indicator Muscle Failure (Pre-Intervention) (2); Post-Functional Neurology Treatment (3); Post-Indicator Muscle Failure (Post-Intervention) (4); Pressure Pain Threshold (PPT), Critical Flicker Fusion Threshold (CFFT). Results are presented as means ± standard deviations. Significant differences (*p* < 0.05) are noted.

## Data Availability

Data are contained within the article.
